# Mean corpuscular volume of control red blood cells determines the interpretation of eosin-5′-maleimide (EMA) test result in infants aged less than 6 months

**DOI:** 10.1007/s00277-015-2377-0

**Published:** 2015-04-25

**Authors:** Olga Ciepiela, Anna Adamowicz-Salach, Weronika Bystrzycka, Jan Łukasik, Iwona Kotuła

**Affiliations:** Department of Laboratory Diagnostics and Clinical Immunology of Developmental Age, Medical University of Warsaw, Marszalkowska 24, 00-576 Warsaw, Poland; Department of Pediatrics, Hematology and Oncology, Medical University of Warsaw, Marszalkowska 24, 00-576 Warsaw, Poland; Students Scientific Group at Department of Laboratory Diagnostics and Clinical Immunology of Developmental Age, Medical University of Warsaw, Marszalkowska 24, 00-576 Warsaw, Poland

**Keywords:** Eosin-5′-maleimide, Flow cytometry, Hereditary spherocytosis, Mean corpuscular volume

## Abstract

Eosin-5′-maleimide (EMA) binding test is a flow cytometric test used to detect hereditary spherocytosis (HS). To perform the test sample from patients, 5–6 reference samples of red blood are needed. Our aim was to investigate how the mean corpuscular volume (MCV) of red blood cells influences on the value of fluorescence of bounded EMA dye and how the choice of reference samples affects the test result. EMA test was performed in peripheral blood from 404 individuals, including 31 children suffering from HS. Mean fluorescence channel of EMA-RBCs was measured with Cytomics FC500 flow cytometer. Mean corpuscular volume of RBCs was assessed with LH750 Beckman Coulter. Statistical analysis was performed using Graph Pad Prism. The correlation Spearman coefficient between mean channel of fluorescence of EMA-RBCs and MCV was *r* = 0.39, *p* < 0.0001. Interpretation of EMA test depends on MCV of the reference samples. If reference blood samples have lower MCV than the patients MCV, EMA test result might be negative. Due to different MCV values of RBCs in infancy and ca. Three months later, EMA test in neonates might be interpreted falsely negative. Samples from children younger than 3 months old had EMA test result 86.1 ± 11.7 %, whereas same samples that analyzed 4.1 ± 2.1 later had results of 75.4 ± 4.5 %, *p* < 0.05. Mean fluorescence of EMA-bound RBC depends on RBC’s volume. MCV of reference samples affects EMA test results; thus, we recommend selection of reference samples with MCV in range of ±2 fL compared to MCV of patient RBC’s.

## Introduction

Hereditary spherocytosis (HS) is an inherited disorder of the red blood cell plasma membrane. The plasma membrane of erythrocytes consists of an outer lipid bilayer and an inner cytoskeleton layer. Subjects with HS are characterized with inherited deficiency of cytoskeleton proteins: band 3, protein 4.2, α-spectrin, β-spectrin, or/and ankyrin [1–6]. To diagnose HS, two main laboratory tests are recommended: eosin-5′-maleimide (EMA) binding assay and cryohemolysis test [3]. Fluorescent EMA dye binds to band-3-protein, and the deficiency of this protein results in decreased fluorescence of the stain. Mean fluorescence of EMA-bound cells is measured by flow cytometry [7–15]. In order to interpret the EMA test, 5–6 normal reference samples should be prepared for every sample from a patient [12]. The exact result of the test is presented as percentage of decrease of EMA fluorescence compared to fluorescence of normal RBC bound with EMA. Most published studies on EMA test and diagnostics of HS were performed on adult subjects [9, 12]. In such cases, choice of normal blood samples from blood donors for reference materials is substantiated. But, the choice of obtaining reference samples from adult blood donors while testing for pediatric patients looks controversial. Recommendation of British Committee for Standards of Hematology emphasizes that laboratory diagnostics of HS should be performed for children older than 6 months old [3]. However, an EMA binding test is commonly performed on neonates and infants younger than 6 months, as a first-line test in HS screening. Considering this, it is imperative that a suggestion of a suitable reference material should be made.

This paper examines the correlation between mean channel of fluorescence of EMA-conjugated RBC and mean corpuscular volume (MCV) of studied cells, to show the influence of reference cell volume on EMA test results in pediatric samples. It is also aimed to propose the most suitable reference blood samples for EMA test performed for neonates and infants.

## Methods

### Analyzed samples

Results of peripheral blood tests performed for 404 children from various departments of the pediatric hospital at the Medical University of Warsaw were taken up for study. The median age of included subjects was 5 years (1 week to 18 years), both boys and girls. Results of children from oncology department were excluded from the study. First group of studied patients consisted of 373 children without HS. Second group consisted of 31 children with confirmed HS. The characterization of the HS subjects is presented in Table 1. All investigated blood samples were part of the specimens previously used for requested routine blood tests—EMA binding test and complete blood count (CBC) analysis.

**Table 1:** Characteristics of HS patients

Patient	Sex	Age	EMA [%]	OF [s]	MCHC [g/dL]	Family history
1	F	7 months	66,1	-	35,3	+
2	F	9 months	71,2	90	35,8	+
3	F	2 months	79,2	70	34,1	+
4	M	17 years	79,1	50	34,7	+
5	M	3 years	65,9	40	33,3	+
6	M	3 years	72,9	-	36,2	+
7	F	6 years	68,9	-	36,1	+
8	M	9 years	73,9	-	34,7	+
9	M	10 years	77,9	-	34,6	+
10	F	1 month	76,9	240	36,4	+
11	F	4 years	54,2	-	34.0	+
12	M	7 years	82	62	35,8	+
13	M	1 months	77,7	-	33,5	+
14	M	4 years	71,9	45	34,4	+
15	F	4 months	77,1	-	34,3	+
16	M	1 month	71,5	-	34,4	+
17	M	2 months	77,1	-	33,8	+
18	F	2 months	80,5	-	35,8	+
19	F	1 month	67,5	-	34,7	+
20	M	1 month	79,9	-	35,1	+
21	F	5 years	79	-	35,2	+
22	M	13 years	70,7	110	36,6	+
23	M	7 years	69,1	-	35,1	+
24	F	12 years	70	-	36,7	+
25	M	2 years	72,3	-	32,8	+
26	F	4 months	63	85	34,9	+
27	M	6 years	59,4	-	33,5	+
28	F	9 years	58,4	-	35,5	+
29	M	7 years	70,4	-	34,6	+
30	M	8 years	63,2	-	34,6	+
31	M	1 month	77,7	-	36,3	+

*EMA* result of eosin-5′-maleimide binding test, *OF* result of osmotic fragility test determined by an acidified glycerol lysis test (if “-” the test was not performed), *MCHC* mean corpuscular hemoglobin content, “*+*” positive family history

A retrospective analysis of EMA test results with regard to mean corpuscular volume of studied red blood cells was performed for nine children (six girls and three boys), who were examined in the department twice: 1–3 months after birth and 3–8 months after first analysis.

Both tests were performed using blood collected into tubes containing EDTA. CBC was analyzed within 2 h after blood collection. EMA test was performed within 72 h of material sampling. Blood for EMA test analysis was stored in 4 °C until testing. Complete blood count result was analyzed with regard to mean corpuscular volume of red blood cells. No other hematological parameters were considered. The study was approved by the Ethics Committee at Medical University of Warsaw.

### Eosin-5-maleimide binding test

The procedure of the test was according to that described previously [16]. Five microliters of whole blood from each sample was washed with 0.9 % NaCl solution and incubated in darkness at room temperature for 1 h with 25 μL of EMA dye (0.5 mg/mL, in phosphate-buffered saline, PBS; Fluka, Gillingham, UK), with intermittent mixing. Stained red blood cells were washed three times using 0.5 %/FBS/PBS and centrifuged after each wash. Cells were suspended in 500 μL of 0.5 % FBS/PBS solution, and 100 μL of labeled cell suspension was diluted in 1.4 mL of FBS/PBS. Flow cytometric analysis was carried out using Cytomics FC 500 flow cytometer (Beckman Coulter, Fullerton, CA, USA). Mean channel of fluorescence (MCF) units were determined in the FL-1 channel for 100,000 events.

The result of an EMA test is presented as a mean channel of fluorescence units (MCF) as well as in the percentage of control fluorescence (%) counted as follows:

$$ \mathrm{Patient}'\mathrm{s}\ \%=\frac{\mathrm{patient}'\mathrm{s}\kern0.5em \mathrm{M}\mathrm{C}\mathrm{F}}{\left(\mathrm{M}\mathrm{C}\mathrm{F}\ 1+\mathrm{M}\mathrm{C}\mathrm{F}\ 2+\mathrm{M}\mathrm{C}\mathrm{F}\ 3+\mathrm{M}\mathrm{C}\mathrm{F}\ 4+\mathrm{M}\mathrm{C}\mathrm{F}\ 5+\mathrm{M}\mathrm{C}\mathrm{F}\ 6\right)\div 6}\ast 100\% $$

where:

Patient’s %: patient’s result
Patient’s MCF: mean channel of fluorescence of stained RBC of patients
MCF 1–6: mean channel of fluorescence of stained RBC of control

The cutoff value for positive result of EMA test in the laboratory is 81 %, as has already been established in a previous study [17].

We did not base the result of EMA test only on mean channel of fluorescence, because the results obtained from different laboratories can vary significantly and are not comparable when analyzing the data from the same patient [18].

The flow cytometer was standardized against Flow-set (Beckman Coulter, USA) fluorospheres.

### Statistical analysis

The Spearman’s rank correlation coefficient was used to analyze correlation between MCV and MCF of EMA. One-way ANOVA test was used for comparison of MCV and MCF from three different reference sample groups. Wilcoxon matched pair test was performed to compare the difference between EMA test results repeated for children at different ages. A *p* value of <0.05 was taken as being significant for the analysis. Results are presented as mean ± standard deviation (SD). Statistical analysis was performed with GraphPad Prism 6 (GraphPad Software Inc., USA).

## Results

### Correlation of MCF and MCV

**Fig. 1:**
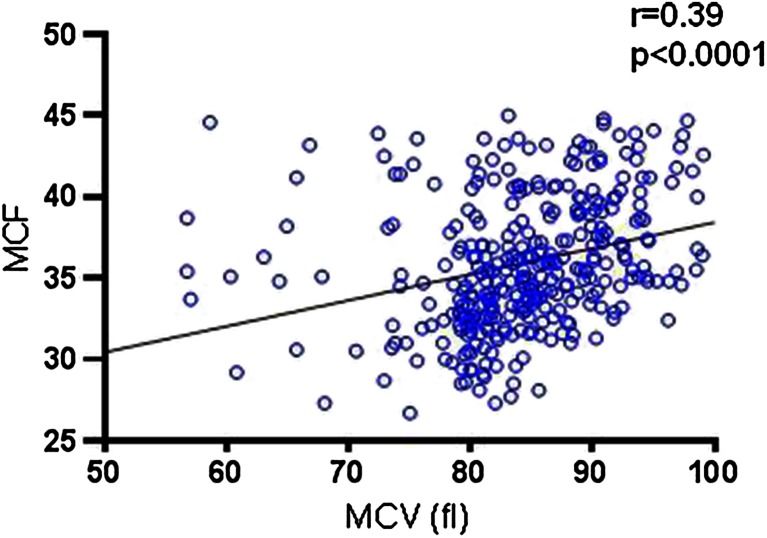
Correlation between mean corpuscular volume (MCV) of red blood cell and mean channel of fluorescence (MCF) of EMA-conjugated erythrocytes, *r* = 0.39, *p* < 0.0001

Correlation between mean corpuscular volume of red blood cells and mean fluorescence channel of EMA-conjugated RBC was analyzed for 383 blood samples. Analyzed samples had MCV values ranging from 48.8 to 106.2 fL (84.6 ± 7.7 fL), whereas values of mean fluorescence were 26.7 to 45.0 mean fluorescence intensity (MFI) units (35.97 ± 3.99 MFI). There was a positive correlation between both analyzed parameters with Spearman coefficient 0.39, *p* < 0.0001 (Fig. 1).

Correlation between mean channel of fluorescence and MCV was also analyzed for 31 blood samples from children suffering from HS. Average MCF of analyzed samples was 27.7 ± 3.3 MFI; meanwhile, MCV was 82.4 ± 6.8 fL, although no correlation between analyzed parameters was found (*p* > 0.05).

### Test interpretation with regard to different MCV values from reference samples

**Fig. 2:**
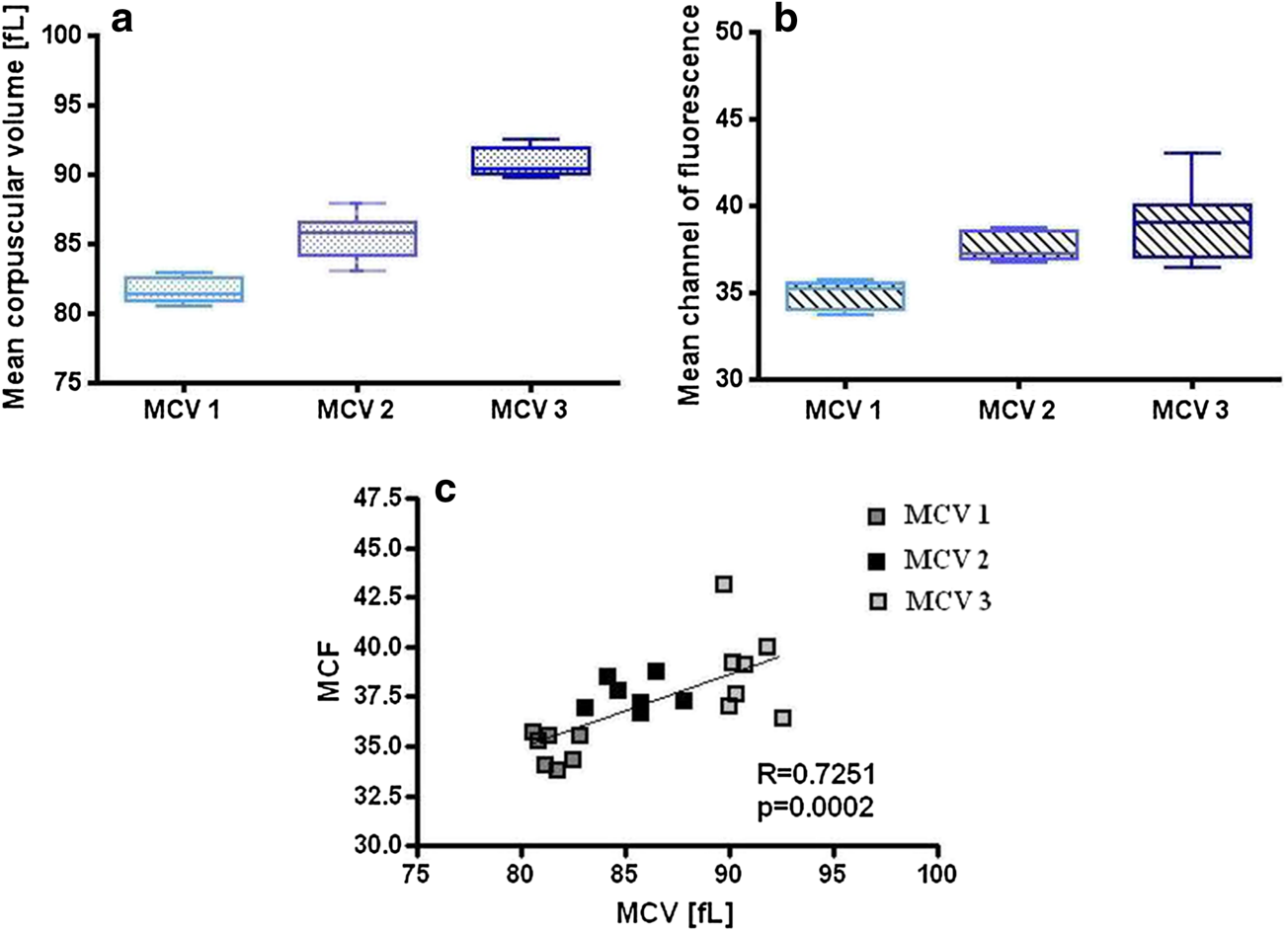
Ranges of mean corpuscular volumes (**a**) and corresponding mean channels of fluorescence (**b**) for reference samples used for the interpretation of EMA test experiment. Correlation of MCF and MCV for analyzed samples (**c**)

To obtain the exact result of EMA test, the mean intense of patient’s dye-conjugated RBC fluorescence should be compared with the average mean fluorescence of EMA-bound RBC of 5–6 healthy controls. Basing on previous results indicating correlation between EMA fluorescence and MCV of studied red blood cells, an analysis of interpretation of the test with regard to different MCV of control samples was performed. For the experiment, 21 control samples were divided into three groups with different MCV of RBC. The values of MCV were as follows: 81.6 ± 0.8 fL for MCV1 sample group, 85.4 ± 1.6 fL for MCV2 sample group, and 90.8 ± 1.0 fL for MCV3 sample group. Mean channel of fluorescence of RBC for all three sample groups was 34.9 ± 0.8 MFI for MCV1, 37.6 ± 0.8 MFI for MCV2, and 38.9 ± 2.2 MFI for MCV3 (Fig. 2). The differences in the mean corpuscular volume of RBC for all three MCV sample groups were statistically significant, *p* < 0.05 for MCV1 vs MCV2, MCV2 vs MCV3, and MCV1 vs MCV3. Difference in mean channel of fluorescence between MCV1 and MCV2 as well as MCV1 and MCV3 was statistically significant, *p* < 0.05. There was no significant difference in MCF between MCV2 and MCV3.

In the same experiment, peripheral blood samples from six patients suffering from HS were stained with EMA dye. Mean corpuscular volume and mean channel of fluorescence of patients’ red blood cells are presented in Table 2 that also presents exact results of EMA test when different control sample groups were used as a reference (MCV1, MCV2, and MCV3).

**Table 2:** Results of MCV, MCF, and EMA test for HS patients with regard to different volume of control red blood cells

Lp	MCV	MCF	Age	Reticulocytes [×10^6^/μl]	EMA result in relation to MCV1 reference samples [%]	EMA result in relation to MCV2 reference samples [%]	EMA result in relation to MCV3 reference samples [%]
1	83.5	28.5	2 m	0.1703 (↑)	**81.7**	75.8	73.2
2	90.9	30.3	17 y	0.1737 (↑)	**86.9**	**80.6**	77.9
3	70.9	25.8	6 y	0.3121 (↑)	74.0	68.6	66.3
4	81.8	26.5	13 y	0.3288 (↑)	76.0	70.5	68.1
5	74.9	25.9	7 y	0.2418 (↑)	74.3	68.9	66.6
6	94.6	28.9	1 m	0.0556 (N)	**82.9**	76.9	74.3

Results which do not confirm diagnosis of hereditary spherocytosis are bolded

*m* months, *y* years, *(↑)* exceeds reference values for age, *(N)* within reference values for age

### The impact of age on EMA test result

To show how the age of a patient influences the EMA test, a comparison between results obtained in 9 infants younger than 3 months old and results of tests repeated after 4.1 ± 2.1 months was performed. There was a statistically significant difference between values obtained in infants younger than 3 months (86.1 ± 11.7 %) and 1–8 months after first analysis (75.4 ± 4.5 %), *p* < 0.05 (Table 3).

**Table 3:** Results of first and follow-up analysis of EMA test in children suffering from hereditary spherocytosis

Patient	First-time analysis			Follow-up analysis		
	Age [months]	Result of EMA test [%]	Mean corpuscular volume of RBCs	Age [months]	Result of EMA test [%]	Mean corpuscular volume of RBCs
1	1.5	76.2	88.2	8	71.2	77.9
2	2	73.8	85.2	5	72.6	78.5
3	2	81.4	83.2	3	75.7	76.8
4	1	90.3	90.8	4	77.1	82.1
5	2	90.9	86.6	6	82.1	80.4
6	1	113.2	105	4	83.0	82.4
7	0.5	79.4	86.0	6	71.8	68.9
8	0.5	85.6	71.8	6	71.8	69.6
9	1	84.5	88.1	7	73.0	77.1

Mean corpuscular volume of analyzed samples was 88.6 ± 6.4 fL (children younger than 3 months) and 77.1 ± 4.0 fL (children aged 4–9 months), respectively, *p* < 0.05. Additionally, ratios between EMA test result and MCV (EMA/MCV) for all children were calculated. Samples collected from children in the first 3 months of their life were characterized by EMA/MCV ratio of 0.97 ± 0.07, samples collected from the same children 3.5 ± 2.3 months after the first test had EMA/MCV ratio of 0.98 ± 0.05. No difference between obtained values was found.

## Discussion

EMA binding test is used as a screening test to diagnose HS [3, 7–16]. The result of the test is calculated as a decrease in the fluorescence of EMA-bound red blood cells of patients compared to EMA fluorescence of six healthy reference samples.

Most of the laboratories doing EMA testing use adult blood donors’ samples as reference specimens in every analysis. However, it is questionable whether this practice is appropriate with regard to the confirmation of diagnosis in infants and newborns suspected of HS.

Even though it is recommended that EMA test be done only in children over 6 months of age [3], most of the blood samples from infants suspected of HS, with family history of HS, or in particular with mean corpuscular hemoglobin concentration (MCHC) of red blood cells suggesting the disease [3] are directed for the HS screening test. Based on our experience, many children suspected of HS in the first days of their life have a negative result in the EMA test, despite having typical symptoms of the disease and the results of complete blood count and biochemical serum analysis being characteristic for hemolytic anemia. However, the EMA test repeated in the next 3–6 months confirms the diagnosis. On the other hand, Christensen et al. suggest that EMA test is the most reliable test to diagnose HS, with a sensitivity that is comparable to values obtained in adults [19].

Early diagnosis of HS, especially in children with significant neonatal jaundice, is necessary to avoid development of kernicterus [19–21]. Neonates with HS often suffer from severe hyperbilirubinemia and should be treated with prolonged phototherapy [20]. Anemia requiring repeated transfusions occurs in the first months of life [3]. A family history of HS is commonly a factor that allows for the introduction of early diagnostic procedures in neonates. But, many cases of HS are de novo mutations, mainly within ankyrin-1 or β-spectrin genes [20]. The choice of diagnostic methods that can be successfully used for HS screening in neonates is limited. Severe hyperbilirubinemia may be associated with other hemolytic anemias, with the possibility of spherocytes appearing in blood smear in the course of neonatal immune hemolytic anemia or microangiopathic anemia [22]. Christensen et al. introduced a “Neonatal HS index” calculated as MCHC/MCV of studied red blood cells. This index was shown to have high negative predictive value with high false positive result number. All studied children had an index of 0.36 or higher [21]. Last reports show that cryohemolysis is not useful, not only in the diagnosis of neonates, but even within HS subjects of every age, especially for distinguishing from other anemias [23]. Finally, EMA binding test, which is highly specific for HS, could be applied as an early diagnostic process in neonates; however, together with complete blood count, result might be unclear [6]. Decreased fluorescence of EMA-bound red blood cells is found in hereditary pyropoikilocytosis, cryohydrocytosis, congenital dyserythropoietic anemia type II (CDA II), or South-East Asian ovalocytosis [6].

The problem of low sensitivity of EMA test in newborns may be associated with the choice of reference samples as a control for the test. The reference values for mean corpuscular volume of red blood cells for children aged 0–1 months are 88–125 fL, while reference values for adults are significantly lower (77–94 fL) [24]. A smaller cell contains less plasma membrane proteins than a cell with bigger volume; thus, comparing RBCs with different volumes may result in false negative results of EMA test.

Here, we prove that the results of EMA test in newborns and infants younger than 3 months of age and suspected of HS are statistically different from the results of the test obtained from the same patients when they were 4–9 months old. For 3/6 of studied subjects, the result of EMA test performed in the first examination did not confirm the diagnosis (EMA test result was higher than 90 %); however, the test repeated after ca. Three months was indicative for HS. It may be associated with the volume of studied cells, since MCV in the first analysis was significantly higher than in the second and the ratio between EMA test result and MCV of tested blood calculated for both analysis did not differ from each other. It is an argument for making a selection of the chosen reference blood samples where the selected probes should have MCV corresponding to the patient’s RBC MCV.

Results of the performed study show that mean fluorescence of EMA dye bound to plasma membrane proteins is strictly connected with MCV of RBC of analyzed samples. A trend showing the association between volume of cell and mean fluorescence of EMA-conjugated RBCs was first shown by King et al. [12]. The correlation between mean channel of fluorescence and mean cell volume was also demonstrated by Lijeholm et al. That study was performed for 31 patients suffering from congenital dyserythropoetic anemia type III (CDA III) [25]. Our results also point to a positive correlation between the analyzed parameters; however, the correlation coefficient is lower than what the Swedish group has indicated [25]. The difference may be associated with the different number of studied samples. The correlation between EMA fluorescence and MCV was also confirmed for healthy adults (15 studied cases), for hereditary elliptocytosis patients (22 studied patients), and for subjects with iron deficiency anemia (IDA) (13 studied patients). No relationship between MCF and MCV was found for subjects suffering from HS and thalassemia minor [10]. Here, we confirm that no correlation between MCF and MCV was found in patients with HS, primarily because a decreased amount of plasma membrane proteins does not affect the cell volume. The association between cell size and EMA fluorescence was also observed in a group of patients suffering from iron deficiency (significantly lower fluorescence compared to healthy controls) or with macrocytosis (significantly higher fluorescence compared to healthy donors) [10], which indicates that changes in MCV values are strictly associated with the intensity of the fluorescence in EMA test. Increased MCF in macrocytic anemia was also proved by Kar et al.; however, they do not observe any influence of low MCV in microcytic anemia on the fluorescence of RBCs in EMA test [11]. On the other hand, some cases of IDA with spectacularly small red blood cells could be false positively diagnosed as a plasma membrane deficiency in EMA test, if reference red blood cells have significantly higher MCV values. In turn, patients with HS suffering also from anemia caused by folate or vitamin B12 deficiency could be falsely diagnosed as negative in EMA test, when MCV of their RBC would be compared with smaller control red blood cells. Bolton-Maggs points to the possible masking role of megaloblastic anemia with regard to HS [3].

Basing on the proved dependence between MCV and MCF, we decided to study how the test result would be modified if samples with different MCV were to be chosen as reference samples in EMA test. Interestingly, if patients suffering from HS with MCV of RBCs bigger than 83 fL were confronted with reference samples of MCV in the range of 80–83 fL, the obtained results of EMA test were negative (>81 %). Test results were not positive till patients’ samples were compared with reference RBC with corresponding values of MCV. It supports our suggestion that during the selection of reference samples, MCV of the selected specimens should be taken into account. In the present paper, we point especially to the group of neonates, since MCV of infants undergo significant changes with age. In most cases of HS in older age, MCV of patients do not diverge from average MCV for adults. Thus, using reference blood samples from blood donors is suitable. However, some HS patients have MCV exceeding the reference range (mainly by increased number of reticulocytes), and it should be taken into consideration when analyzing EMA test results.

To conclude, EMA test results strongly depend on fluorescence of EMA-bound reference samples used in analysis. Normally, blood from healthy adult blood donors is used as a reference. It is only suitable if the test is performed for adult patients suspected of HS and if their MCV do not exceed reference values. If the studied sample is obtained from a child, especially a newborn, a selection of reference samples with corresponding MCV values should be taken into consideration. Reference samples must be collected from subjects with no anemia, with normal number of red blood cells and without any diseases which could affect the complete blood count result. Based on our observations, we strongly recommend that peripheral blood samples with MCV differing from the patient’s RBC MCV by ±2 fL should be used as reference.
